# Lister and the Spray

**Published:** 1883-09

**Authors:** 


### Lister and the Spray.

Dr. Henry Gray Croly publishes the following letter from Mr. Lister on this subject in the *Medical Press*, April 25, 1883:

"I have not given up the use of the spray, although I certainly regard it as the least important part of our antiseptic arrangements. Whatever other good it may do, it is a very mild form of antiseptic irrigation, and tends to keep the *entourage* of the wound, including the surgeon's hands and instruments, pure. But if I had not a spray-producer at hand, I should not on that account omit other elements of antiseptic treatment. I still use the spray in changing dressings, so long as the wound is not merely superficial. But far more important than using the spray is it to make a point of covering the wound with some pure aseptic material before beginning to wash the parts which were covered with the edge of the dressing only, and were, therefore, impure. In other words, I believe one of the commonest causes of failure is dabbing alternately the impure surrounding parts and the pure wound with the same piece of rag, which, though moistened with carbolic lotion, cannot work miracles."

